# A New Model for Solving Time-Cost-Quality Trade-Off Problems in Construction

**DOI:** 10.1371/journal.pone.0167142

**Published:** 2016-12-02

**Authors:** Fang Fu, Tao Zhang

**Affiliations:** School of Economics & Management of China University of Petroleum (East China), Qingdao, Shandong, PR China; West Virginia University, UNITED STATES

## Abstract

A poor quality affects project makespan and its total costs negatively, but it can be recovered by repair works during construction. We construct a new non-linear programming model based on the classic multi-mode resource constrained project scheduling problem considering repair works. In order to obtain satisfactory quality without a high increase of project cost, the objective is to minimize total quality cost which consists of the prevention cost and failure cost according to Quality-Cost Analysis. A binary dependent normal distribution function is adopted to describe the activity quality; Cumulative quality is defined to determine whether to initiate repair works, according to the different relationships among activity qualities, namely, the coordinative and precedence relationship. Furthermore, a shuffled frog-leaping algorithm is developed to solve this discrete trade-off problem based on an adaptive serial schedule generation scheme and adjusted activity list. In the program of the algorithm, the frog-leaping progress combines the crossover operator of genetic algorithm and a permutation-based local search. Finally, an example of a construction project for a framed railway overpass is provided to examine the algorithm performance, and it assist in decision making to search for the appropriate makespan and quality threshold with minimal cost.

## Introduction

A construction project is scheduled with the constraints of time, cost, and quality, where crashing or excessive saving may lower project quality. This scenario is the time-cost-quality trade-off problem. However, repair works may be adopted to attain the standard requirements during construction, depending on the inspection of the quality established by a set of activities, but repair works would increase project makespan and total cost. Thus, the entire process should be modelled to describe the complete relationship among time, cost and quality.

The time-cost-quality trade-off problem has received increasing attention recently due to the significance of quality. Objective functions are often involved with project quality, which is expressed as the average or minimum activity quality. The minimum activity quality is used to depict the weakness of the project [1–2], whereas the average is employed to replace project quality with intermediate value of activity quality [3]. Besides, a linear combination method of the average and minimum is presented by allocating weights in advance [4]. However, this depiction or replacement is not always appropriate, especially for project networks with complex structures. In the paper, we search for the minimum quality cost by Quality-Cost Analysis [5] to avoid measuring project quality.

The research can be divided into two groups, classified by the method for handling activity quality. In the first group, many multi-criterion approaches, from the analytical hierarchy process (AHP) to multi-objective programming, are employed after the prediction of all activity qualities [6–10]. However, one of the most difficult techniques is the detection and estimation of the activity qualities before project scheduling; therefore, another method is presented based on the bids from many specialists or the scenarios from previous materials to address activity qualities. Babu and Suresh (1996) and Khang and Myint (1999) applied the linearity assumption between quality and time, where the quality associated with all normal times was set to 1.0 and the lower quality at the crash time for each activity was 0 [11–12]. However, the linearity assumption between quality and time was considered to be not always in effect. Kim et al. (2012) considered the potential quality loss cost for excessive crashing activities and proposed a mixed integer linear programming model, but the assumption that crashing leads to potential quality loss cost is not always realised [13]. Moreover, there is another way to prevent the linearity assumption: Salmasnia et al. (2012) incorporated non-linear stochastic programming into the trade-off problem to minimize the variation effect on time, cost, and quality, but it is controversial to believe that any change to the resource input must influence quality [1]. Liberatore and Pollack-Johnson (2013) introduced a bivariate normal function of time and cost to describe task quality and conducted estimation and data fitting for two case studies to maximize the minimum activity qualities [14]. However, the assumption that activity duration is independent of cost may not be practical for construction projects. In the paper, we cancel the assumption and rebuild a non-linear correction function to describe activity quality (Section 2.1).

In conclusion, a non-linear programming model is presented based on two assumptions to extend the time-cost-quality trade-off problem: activity quality is described as a non-linear correction function of activity duration and its costs; the occurrence of repair work depends on the cumulative quality, which is the quality of a given set of finished activities. Moreover, the multi-constraint optimization model [15] minimizes the project quality costs to search for an appropriate project quality and resource allocation [16] according to Quality-Cost Analysis. The remainder of this paper is structured as follows. Section 2 illustrates the quality function presented in the paper, mainly with regard to activity quality and project quality functions. The non-linear programming model is developed in Section 3. Section 4 describes an adaptive shuffled frog-leaping algorithm. Section 5 develops an illustrative example to study the effectiveness. Finally, conclusions and future works are presented.

## Quality Function

Two types of qualities are defined in the paper. Activity quality, as a work quality, is the degree to which an activity protects the product quality. Cumulative quality, as a product quality, is the degree to which a set of finished activities fulfils requirements, and is expressed by activity qualities.

### Activity quality

The activity quality function is typically an increasing function of activity duration and direct cost, and activity duration is not independent of its direct cost because the duration can be influenced by various resource allocations and the direct cost. Thus, two common functions satisfy the requirement of activity quality, namely, the binary normal function and binary logic function [14]. We choose the former and evaluate it by data fitting. Therefore, activity quality *q* in the paper is described to be a non-linear function as Eq 1 shown:

$$q=\rho e^{-\frac{1}{2\left(1-\eta^{2}\right)}\left[{\left(\frac{t-\mu_{t}}{\sigma_{t}}\right)}^{2}-2\eta \left(\frac{t-\mu_{t}}{\sigma_{t}}\right)\left(\frac{c-\mu_{c}}{\sigma_{c}}\right)+{\left(\frac{c-\mu_{c}}{\sigma_{c}}\right)}^{2}\right]}$$ (1)

Where the activity quality *q* is designed as a function of its duration *t* and direct cost *c*; *μ*_*t*_ and *σ*_*t*_ denote the expectation and standard deviation of the activity duration, respectively; *μ*_*c*_ and *σ*_*c*_ are the expectation and standard deviation of the direct cost, respectively; *η* is a intrinsic parameter in a range of 0–1 to adjust the surface shape, and *ρ* tends to restrict the scope of the activity quality to simplify the computation.

The data fitting is conducted by the MATLAB curve fitting toolbox to validate the activity quality function. For example, 6 scenarios for an activity, “pit excavation”, from previous materials involving time, cost, and quality (as shown in Table 1, details in section 5.2) have been investigated. The six parameters (*η*, *ρ*, *μ*_*t*_, *μ*_*c*_, *σ*_*t*_ and *σ*_*c*_) in the bivariate normal function are determined using nonlinear least squares estimation by the curve fitting toolbox automatically. Given that the maximum duration and direct cost are 6 days and $1544.2, respectively, and that the activity quality ranges from 0 to 1, the activity quality with a duration of 4 days and a direct cost of $1345 is obtained. The other results are presented in Table 2, in which the SSE (Sum of Squared Error) and R^2^ indicate that the data fitting is relatively acceptable.

**Table 1:** Duration, cost and quality for pit excavation.

No.	Duration (days)	Direct cost ($)	Quality (0–1)	No.	Duration (days)	Direct cost ($)	Quality (0–1)
1	5	1249.6	0.89	4	4	1510.2	0.93
2	4	1324.5	0.90	5	2.5	1383.6	0.87
3	4	1443.3	0.92	6	2.5	1399.7	0.90

**Table 2:** Data fitting results.

SSE	R^2^	*η*	*ρ*	*μ*_*t*_	*μ*_*c*_	*σ*_*t*_	*σ*_*c*_	activity quality
0.03	0.87	0.99	0.95	6	1544.2	63.14	725.0	0.89

### Cumulative quality

Cumulative quality is tracked as a certain part of project quality to trigger repair or rework. Measurement and inspection are conducted in stages during construction, and if the outcome does not satisfy the basic requirements, repair or rework is adopted to remedy the defects. Therefore, cumulative quality is designed to express the quality of finished activities under the present conditions.

However, the activities differ in their contribution to the project quality. For example, both foundation and wall plastering affect the quality of a building; but the foundation is more important than plastering for determining project quality because the former is crucial to the reliability and stability of the building.

**Fig 1:**
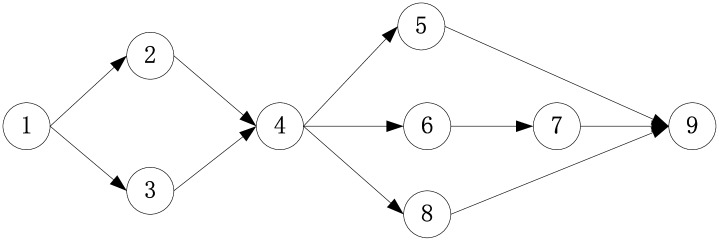
Activity-On-Node network.

For example, a small construction project with an Activity-On-Node (AON) network is shown Fig 1 and Table 3, where all of the activity qualities ranging from 0 to 1 are generated by the method in Section 2.1. If an activity quality exceeds 0.8, the activity is generally regarded to attain the standards. One of three alternatives in Table 3 will be selected as the final construction process for each activity, and the 3^rd^ set of construction processes is generally favoured in practice.

**Table 3:** Activity qualities of three sets of alternative construction processes.

No. of group	No. of activity	Activity	Activity quality of 1^st^ construction process	Activity quality of 2^nd^ construction process	Activity quality of 3^rd^ construction process
group 1	2	Precast pile	1	0.6	0.8
	3	Precast roof truss	0.8	0.6	0.8
	4	lifting	0.6	0.6	0.8
group 2	6	Roof levelling	1	0.8	0.8
	7	3-ply built-up roof	0.6	0.6	0.8

We need to measure cumulative qualities of two groups: group 1 consists of the activity 2, activity 3 and activity 4; the activity 6 and activity 7 also form the group 2. In Table 4, three approaches for the groups are employed: minimum value of the activity qualities, average value of the activity qualities, and the approach in this paper. Table 4 indicates that if the cumulative quality is depicted as the minimum of activity quality, the 1^st^ and 2^nd^ set of construction processes are identical; if the average value denotes the cumulative quality, the 1^st^ and 3^rd^ set of construction processes both will be selected. However, the approach in the paper could differ the three processes and choose the right one.

**Table 4:** different approach for cumulative quality.

No. of group	approach	cumulative quality of 1^st^ construction process	cumulative quality of 2^nd^ construction process	cumulative quality of 3^rd^ construction process
group 1	Minimum	0.6	0.6	0.8
	Average	0.8	0.6	0.8
	This paper	0.54	0.36	0.64
group 2	Minimum	0.6	0.6	0.8
	Average	0.8	0.7	0.8
	This paper	0.6	0.48	0.64

**Fig 2:**
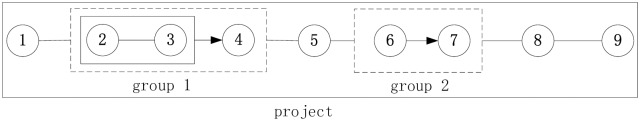
Analysis of the project.

The three construction processes differ in their effect on the cumulative quality in the paper because two relationships among activity qualities are distinguished: precedence relationships and coordinative relationships. The activities in a precedence relationship (PR) that are regarded as prerequisites for the entire, for example, high qualities of *Precast pile* and *Precast roof truss* with poor *lifting* in group 1 would not improve the project quality, and vice versa; in group 2 an uneven screed-coat will cause the waterproof layer to crack, whereas an irregular waterproofing makes high-quality roof levelling meaningless. Second, a coordinative relationship (CR) also exists among these activities, such as the 2^nd^ and 3^rd^ activity for the quality of the group 1. Thus, the project should be decomposed and analysed as shown in Fig 2, where the dashed box and solid box denote coordinative and precedence relationships, respectively.

Furthermore, the computation method of project quality based on activity qualities varies with the different relationships. The activities bonded by a coordinative relationship are described by the average, whereas the activities combined by a precedence relationship are formulated in terms of the product. Thus, if *q*_*i*_ signifies the quality of the *i*th activity, the cumulative qualities of the two groups are computed to be $\frac{\left(q_{2}+q_{3}\right)}{2}\times q_{4}$ and *q*_6_ × *q*_7_ respectively.

Thus, cumulative quality is determined by Eq 2 in conclusion. The activities bonded by a coordinative relationship are described by the average [10], whereas the activities combined by a precedence relationship are formulated in terms of the product by reference to conditional probability, which is similar to the parallel system in reliability theory [17].

$$\prod_{j\in D_{g}}\left(q_{j}\times \frac{1}{NI_{g}}\sum_{i\in ID_{g}}q_{i}\right)$$ (2)

where *D*_*g*_ denotes the *g*th (*g* ∈ {1,2,⋯,*G*}) group to measure cumulative quality, and *ID*_*g*_ shows *NI*_*g*_ activities combined by coordinative relationships within the group *D*_*g*_.

## Non-Linear Programming Model

The model is mainly presented based on the classic multi-mode resource constrained project scheduling problem [18]: A construction project can be described with an activity set, denoted as *ψ* = {0,1,⋯,*N*,*N*+1}, and activities 0 and *N*+1 represent a dummy source and dummy sink activity, respectively. The precedence relationships of activities are finish-start with zero lag time and no pre-emption. *PA*_*j*_ describes the set of immediate predecessors of activity *j*, and the deadline of the project is *T*. The duration of activity *j* executed in mode *m* (*m* ∈ {1,2,⋯,*M*}) is denoted by *da*_*jm*_. There are *L* renewable resources and *K* non-renewable resources. The availability of each type of renewable resource *l* in each time period is *R*_*l*_ with unit cost *CR*_*l*_, whereas the availability of each type of non-renewable resource *k* for the entire project is *N*_*k*_ with unit cost *CN*_*k*_. Each activity *j* executed in mode *m* requires *r*_*jml*_ units of renewable resource *l* (such as staff and machines) and *n*_*jmk*_ units of non-renewable resource *k* (such as materials). Besides, the repair work with a duration denoted by *rd*_*j*_ consumes *rr*_*jl*_ units of renewable resource *l* and *rn*_*jk*_ units of non-renewable resource *k*.

Assuming that repair work is initiated once the cumulative quality of some activities is below the given threshold *δ*, the other specific parameters are presented as follows: *D*_*g*_ is denoted as the *g*th set of dependence relationships, and the last activities of all groups compose a collection described as *A*. *ID*_*g*_ indicates the group of coordinative relationships formed by *NI*_*g*_ activities in the *g*th group of the dependence relationships.

1. Decision Variables
  *x*_*jmt*_ = 1 if activity *j* executed in mode *m* starts at time *t*, 0 otherwise.
  *z*_*jt*_ = 1 if repair work for activity *j* (*j* ∈ *A*_*g*_) starts at time *t*, 0 otherwise.
2. Intermediate Variables
  *q*_*j*_ quality of activity *j* is a binary normal function of the duration and direct cost, which is not permitted to be inferior to a given threshold *δ*.
  *cq*_*j*_ cumulative quality after non-dummy activity *j* is completed.
  *E*_*t*_ *E*_*t*_ = {(*j*,*m*)|*x*_*jm*,*t*−*f*_ = 1, 0 ≤ *f* < *d*_*jm*_}, i.e., a set of activities with their modes that are in progress at time *t*.
  $E_{t}^{\prime }$ $E_{t}^{\prime }=\left\{j|z_{j,t-f}=1,\;j\in A,\;0\leq f<rd_{j}\right\}$, i.e., a set of repair works that are in progress at time *t*.
  *ft* actual completion time of project, i.e., $ft=\sum_{0}^{T}t\times x_{N-1,0t}$.
  *pq* project quality.
3. Objective Function
  In order to maintain tradeoffs among project quality, makespan and total costs, the model minimizes the total quality cost in order to search for a satisfactory project quality level based on quality-cost analysis. The total quality cost denoted as *TC* comprises prevention cost (the difference between the actual direct cost and minimum direct cost of all the activities) and failure cost (namely repair cost during construction). Provided that the direct cost of each activity increases as its mode ranges from 1 to *M*, the objective function is proposed as Eq 3 shown:
  $$\text{Min}:\;TC=C_{j}-C_{j0}+\sum_{l=1}^{L}\sum_{t=0}^{ft}\sum_{j\in a}^{}CR_{l}z_{jt}rr_{jl}rd_{j}+\sum_{k=1}^{K}\sum_{j\in A}^{}CN_{k}z_{jt}rn_{jk}$$ (3)
  where the actual direct cost and minimum direct cost of activity *j* are denoted as *C*_*j*_ and *C*_*j*0_ respectively:
  $$\begin{array}{l} C_{j}=\sum_{l=1}^{L}\sum_{t=0}^{ft}\sum_{j=1}^{N}CR_{l}\sum_{m=1}^{M}r_{jml}x_{jmt}d_{jmt}+\sum_{k=1}^{K}\sum_{j=1}^{N}CN_{k}\sum_{m=1}^{M}n_{jmk}y_{jm}\\ C_{j0}=\sum_{l=1}^{L}\sum_{t=0}^{ft}\sum_{j=1}^{N}CR_{l}r_{j0l}d_{j0t}+\sum_{k=1}^{K}\sum_{j=1}^{N}CN_{k}n_{j0k} \end{array}$$
4. Constraints
  $$\sum_{t=0}^{ft}\sum_{m=1}^{M}x_{jmt}=1\;j=\text{1},\text{2},\ldots ,N$$ (4)
  $$\sum_{t=0}^{ft}\sum_{m=1}^{M}\left(t\times x_{jmt}\right)\geq \sum_{t=0}^{ft}\sum_{m=1}^{M}\left(t-d_{im}-\sum_{\begin{array}{l} t=0\\ i\in A \end{array}}^{ft}\left(z_{it}\times rd_{i}\right)\right)\times x_{imt}\;i\in PA_{j}$$ (5)
  $$\sum_{t=0}^{ft}\left(t\times z_{it}\right)\geq \sum_{t=0}^{ft}\sum_{m=1}^{M}\left(t-d_{im}\right)\times x_{imt}\;i\in A\;\&\;cq_{i}<\delta$$ (6)
  $$\sum_{j\in E_{t}}^{}\sum_{m=1}^{M}r_{jml}+\sum_{j\in E_{t}^{\prime }}^{}rr_{jl}\leq R_{l}\;l=\text{1},\text{2},\ldots ,L$$ (7)
  $$\sum_{j=1}^{N}\left(\sum_{m=1}^{M}\left(n_{jmk}\sum_{t=0}^{ft}x_{jmt}\right)+\sum_{\begin{array}{l} t=0\\ j\in A \end{array}}^{ft}rn_{jk}z_{jt}\right)\leq N_{k}\;k=\text{1},\text{2},\ldots ,K$$ (8)
  $$x_{000}=1$$ (9)
  ft≤T (10)
  $$cq_{j}=\{\begin{array}{ll} \prod_{j\in D_{g}}\left(q_{j}\times \frac{1}{NI_{g}}\sum_{i\in ID_{g}}q_{i}\right) & \sum_{t=1}^{ft}z_{jt}=0\&j\in A\\ \delta & \sum_{t=1}^{ft}z_{jt}=1\&j\in A \end{array}$$ (11)

Eq 4 allows only one mode to be implemented to achieve a definite duration for each activity. Eq 5 illustrates that each activity should start after the immediate predecessors and their relative repair works have been completed. Eq 6 specifies that if the cumulative quality level after a completed non-dummy activity is inferior to the given threshold *δ*, repair work should start immediately. Eqs 7 and 8 are the constraints related to resource availability, i.e., the requirement for renewable resources in each time period and the requirement for non-renewable resources over the project span are below the corresponding availabilities. Eqs 9 and 10 require that the project starts at time 0 and is completed before the deadline. Eq 11 explain the expression of the cumulative quality and project quality level, and if there is repair work, the cumulative quality would be up to *δ*.

## Shuffled Frog-Leaping Algorithm

The classic multi-mode resource constrained project scheduling problem is just NP-hard, and considering cumulative quality and repairs in the paper makes it more complicated. Thus we have to choose meta-heuristics to obtain a satisfactory solution, such as the shuffled frog-leaping algorithm (SFLA). SFLA has been effectively applied to the job-shop scheduling problem [19], vehicle routing problem [20] and outperforms some classical algorithms efficiently and effectively for the resource-constrained project scheduling problem [21]. Thus, we present a hybrid SFLA in the paper.

### Basic shuffled frog-leaping algorithm

SFLA was proposed to solve discrete decision problems by combining the features of the genetic-based memetic algorithm and social behaviour-based particle swarm optimisation [22]. Inspired by the process that frogs use to hunt for food in nature, SFLA is based on the evolution of memes carried by the interactive individuals and a global information exchange among themselves. In the SFLA, the initial population is composed of a given number of randomly generated solutions called virtual frogs. Then, the initial population is divided into a predefined number of parallel subsets referred to as memeplexes, and different memeplexes undergo the evolution process independently. During evolution, the worst frog in each memeplex, denoted as *P*_*W*_, is improved by learning from either the best solution in the memeplex or the best individual of the entire population. Within each memeplex, it is assumed that *P*_*B*_ and $P_{B}^{*}$ are the best solutions of the memeplex and population, respectively. The worst frog after evolution $P_{W}^{\prime }$ is calculated as follows:

$$P_{W}^{\prime }=P_{W}+\{\begin{array}{c} \text{min}\left\{\text{int}\left[rand\times \left(P_{B}-P_{W}\right)\right],S_{m}\right\}\text{ for a positive step}\\ \text{max}\left\{\text{int}\left[rand\times \left(P_{B}-P_{W}\right)\right],-S_{m}\right\}\text{ for a negative step} \end{array}$$ (12)

$$P_{W}^{\prime }=P_{W}+\{\begin{array}{c} \text{min}\left\{\text{int}\left[rand\times \left(P_{B}^{*}-P_{W}\right)\right],S_{m}\right\}\text{ for a positive step}\\ \text{max}\left\{\text{int}\left[rand\times \left(P_{B}^{*}-P_{W}\right)\right],-S_{m}\right\}\text{ for a negative step} \end{array}$$ (13)

where *rand* is a random number between 0 and 1 and *S*_*m*_ is the maximum leaping step size.

If the new frog is better than the old one, then it replaces the old frog. Otherwise, the worst frog is replaced by a randomly generated frog. Finally, all memeplexes are combined, and the frog-leaping and shuffling processes are repeated until a given stopping condition is satisfied.

### Hybrid SFLA

**Fig 3:**
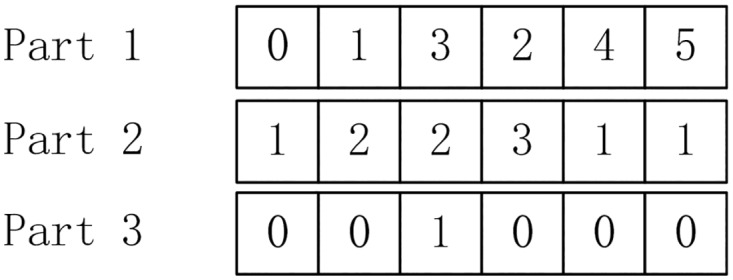
An example of coding.

The SFLA is modified so that each frog or solution is encoded in three parts, namely, an activity list, a mode set and a binary variable set pertinent to each activity. The set of binary variables depends on the relative activity and executed mode, among which the last variable indicates whether a quality accident occurs and the others show whether repair work is conducted for the corresponding activity (it defaults to 0). For example, the coding in Fig 3 shows that the modes of activities 0, 1, 3, 2, 4 and 5 are selected to be 1, 2, 2, 3, 1 and 1, respectively, and activity 3 needs to be repaired.

#### Population initialisation

To improve the entire search efficiency, the initial population is generated through a priority rule-based heuristic and consists of *multi*×*H* solutions. In addition, *multi* and *H* are defined parameters, and *multi* typically ranges from 2 to 10. In the process of building a new solution, the heuristic is used to compute selection probability and to choose one activity or mode based on a roulette wheel. The probability to select a feasible activity and mode is described as Eqs 14 and 15, respectively.

$$P_{LST}\left(i\right)=\frac{\frac{1}{lst_{i}}}{\sum_{i\in \sigma }\frac{1}{lst_{i}}}$$ (14)

$$P_{\text{mod}e}\left(m\right)=\frac{\sum_{l=1}^{L}n_{i*ml}+\sum_{k=1}^{R}r_{i*mk}}{\sum_{m=1}^{M}\left(\sum_{l=1}^{L}n_{i*ml}+\sum_{k=1}^{R}r_{i*mk}\right)}$$ (15)

where *σ* is the set of feasible activities, which are unscheduled but whose predecessors are scheduled. Then, parts 1 and 2 of the solution are obtained, and part 3 is found by Eq 11.

Moreover, the serial schedule generation scheme is adapted to complete feasible schedules, which settle the earliest available start times *s*_*j*_ and finish time *f*_*j*_ for each activity *j* executed in the given mode and according to the activity list sequentially. Part1(*g*), part2(*g*) and part3(*g*) denote the activity, mode and binary variable located at the *g*th position of the solution, respectively. The Pseudo-code of the procedure is presented as follows:

Initialisation: *g* = 0, *πR*_*lt*_ = *R*_*l*_,*πN*_*k*_ = *N*_*k*_ (*t* = 0,1,…,*T*, *l* = 1,2,…,*L*, *k* = 1,2,…,*K*);

While *g* <= *N* + 1 Do

 Begin

  *j* = part1(*g*);

  *m* = part2(*g*);

  *ES*_*j*_ = max{*f*_*i*_|*i* ∈ *PA*_*j*_}+part3(g)×*rd*_*j*_;

  *s*_*j*_ = min{*t*|*ES*_*j*_ ≤ *t*, *r*_*jmr*_ ≤ *πR*_*rτ*_, *n*_*jmk*_ ≤ *πN*_*k*_, *τ* = *t*, ⋯, *t* + *d*_*jm*_ −1;*r* = 1, ⋯, *R*};

  *f*_*j*_ = *s*_*j*_ + *d*_*jm*_;

  Compute current resource availability *πR*_*lt*_ and *πN*_*k*_ considering part3(*g*);

  *g* = *g* + 1;

 Endwhile

Stop

#### Selection

Then, a selection operator is added to select *H* solutions from the initial population to compose the “frog colony”. In the process of the operator, the elitist strategy and “max-min distance” are employed to inherit the top schedule and maintain the diversity simultaneously. The latter is organised as follows: if the distances from the *j*th solution in the initial population to all solutions in the colony are obtained, the minimum is denoted as $dist_{\text{min}}^{j}$; after all $dist_{\text{min}}^{j}$ of the solutions in initial population are achieved, *dist*_max_ is employed to indicate the maximum value. Its corresponding solution is then transferred to the frog colony. The process continues until the given amount of solutions *H* is satisfied. In addition, the distance *dist*(*a*,*b*) between solutions *a* and *b* for the project scheduling problem is defined as in Eq 16, in which $S_{i}^{a}$ and $S_{i}^{b}$ represent the start time of activity *i* in solutions *a* and *b*, and $m_{i}^{a}$, $m_{i}^{a}$ indicate the selected mode of activity *i* in solutions *a* and *b*, respectively.

$$dist\left(a,b\right)=\sum_{i=1}^{N}{\left|s_{i}^{a}-s_{i}^{b}+1\right|}^{\left|m_{i}^{a}-m_{i}^{b}+1\right|}$$ (16)

To highlight the influence of the start time on the distance, the formula is presented in the form of power. In addition, the base and exponential part are incremented by one to prevent an irrational base number or a constant distance.

#### Population partition

The selected population, which consists of *H* frogs, is partitioned into *N*_*m*_ memeplexes, where each frog experiences the evolution process independently. First, all frogs are evaluated by the objective function and sorted in descending order according to the objective value. The rank ((*i* −1)×*N*_*m*_ + *j*) frog enters the *j*th memeplex (*i* = 1,2,…,$\frac{H}{N_{m}}$; *j* = 1,2,…,*N*_*m*_). For example, assuming *N*_*m*_ = 2, the rank 1 and 3 frogs enter the 1^st^ memeplex, and the rank 2 and 4 frogs form the 2^nd^ memeplex.

#### Frog-leaping process

The crossover operator of the genetic algorithm and permutation based local search are combined in the frog-leaping process to inherit contiguous genes from both parents, namely, the best frog (donator) and worst frog (receiver).

Firstly, two random integers ranging from 2 to *N*-1 are generated, and parts 1 and 2 of the genes (namely, activities and modes) located at and between the two numbers are copied to the offspring from the donator. The set of genes is called a block, and part 1 of the genes is called the block activities.

Second, the locations with the minimum and maximum indexes that include the block genes are identified in the receiver. Parts 1 and 2 of the genes located before the minimum index are preserved for the offspring. The activities located between the minimum and maximum indices are screened sequentially, and if it is a non-scheduled predecessor or successor of any block-activity, it and the relevant mode are inherited. Parts 1 and 2 of the genes located after the maximum index are also preserved.

Third, if any unscheduled activity exists, it is located between the minimum and maximum index of the receiver and non-predecessor or non-successor of any block activity; thus, it is located before or after the block with a likelihood of 50%. Then, parts 1 and 2 of the offspring are determined. The entire solution is obtained by the adapted serial SGS.

**Fig 4:**
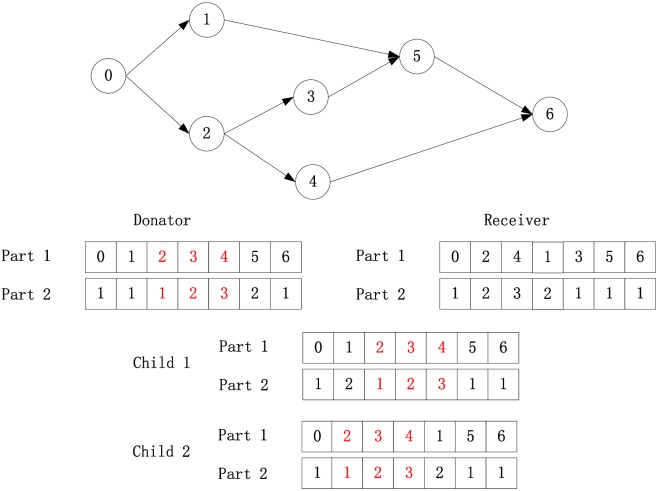
An example of crossover.

Fig 4 shows that with a definite AON network and two given parents, two children are generated with a likelihood of 50%. The red block is copied into both of children, and the precedence among activities and the modes of unblock genes in the receiver are also maintained. Only activity 1 satisfies the requirements to be treated as a predecessor or successor of the block activities; thus, it is likely to be scheduled before or after the block.

Finally, a permutation-based local search is proposed to explore the neighbourhood of an individual to enhance the exploration effect. The local search is performed by varying the mode at a randomly given position. For example, if the mode values range from 1 to 5, when the present mode is 2, it may be adjusted to 1 or 3, and if the present mode is 5, it can only change to 4.

## Example

### Example description

The example is a construction project of a framed railway overpass composed of sixteen activities. The non-dummy activities of the project are executed in one of three modes, only considering 7 types of resources involved with the project quality in the process of execution to simplify computation. The relevant data with respect to the project network and activity information are presented in Table 5 and S1 Table, respectively. The unit costs of the clam shell excavator, hoisting jack and steel bar processing machinery are $85.34, $1.82 and $5.63 per machine-team, the unit cost of a skilled worker and general worker are $38.63 and $20.09 per man-day, and the unit cost of bitumen membranes with 2 mm and 3 mm thicknesses are $3.09 and $4.02 per square meter, respectively.

**Table 5:** Relevant data with respect to the project network.

No.	Activity	Immediate predecessors
0	start	----
1	pit excavation	0
2	manufacturing sliding plates	1
3	lubricating layer and segregation board	2
4	processing reinforcing steels	0
5	formwork fabrication	0
6	precast box-culvert bridge	3
7	box-culvert maintenance, form stripping and waterproofing	4,5,6
8	construction of trust wall	1
9	installation of jacking equipment	7
10	commissioning and idling	9
11	roadway reinforcement	9
12	jacking box-culverts	8,10,11
13	roadway recovery	12
14	appurtenant work	13
15	end	14

### Activity quality and cumulative quality

According to the “Code for construction and quality acceptance of bridge works in city (CJJ2-2008)” in China, the quality acceptances for inspection lots and subprojects are conducted by total inspection or sampling inspection to determine whether they are qualified. The inspection items of each inspection lot or subproject consist of dominant items and general items. The inspection lot or subproject is qualified if the acceptability of each dominant item is 100%, the acceptability of each general item surpasses 80% and the maximum deviation is inferior to 1.5 times the permissible value; otherwise, it is identified as unqualified. Thus, the gaps among the qualified activities can be identified by the average acceptability of the general items, and the activity is unqualified only if the level of an activity quality is equal to or less than 0.8. Thus, the activity qualities in the paper are denoted by the average acceptability of the general items, whereas the quality of any unqualified activity is 0.8.

According to the data fitting conducted by the MATLAB curve fitting toolbox (section 2.1), the results for the activity qualities in each mode range from 0 to 1. All activity qualities after processing are shown in S2 Table, which serves as the input data of the model.

**Fig 5:**
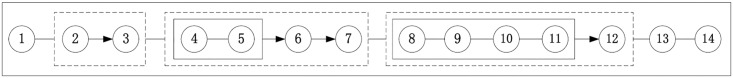
Analysis of the construction project.

The cumulative quality function is obtained by analysis of the relationships among the activities. As shown in Fig 5, there are three dependence relationships among these non-dummy activities where a coordinative relationship may also exist. Besides, the repair durations and requirements for various resources of the 3^rd^, 7^th^ and 12^th^ activities are in Table 5. Thus their cumulative qualities are *q*_2_ × *q*_3_, $\frac{q_{4}+q_{5}}{2}\times q_{6}\times q_{7}$, and $\frac{q_{8}+q_{9}+q_{10}+q_{11}}{4}\times q_{12}$ respectively.

### Result and Analysis

The hybrid SFLA is programmed in Visual C++ 6.0. All the parameters are provided in Table 6 where the maximum iteration *niter* is used as the termination criterion. After computation, the optimal schedule has a total cost of $31135, which includes none failure cost, with a time span of 95 days.

**Table 6:** Algorithm parameters.

*T*	*δ*	*H*	*seg*	*multi*	*niter*
130	0.85	50	3	30	60

**Fig 6:**
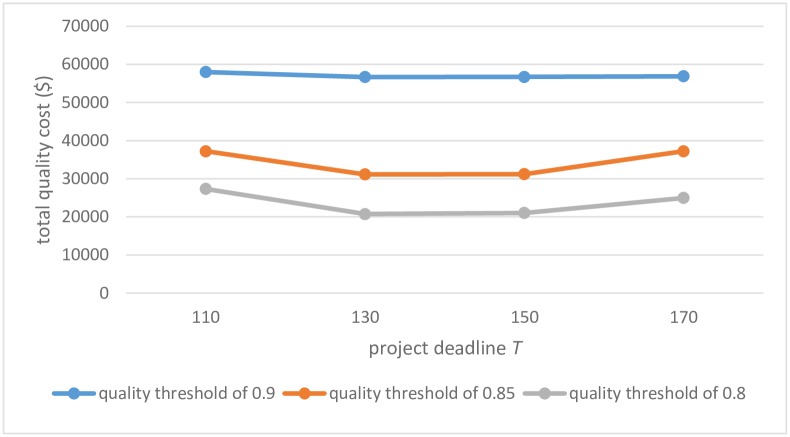
Comparison of total quality costs.

Furthermore, the optimal total cost is presented in Fig 6 for various project deadlines *T* and cumulative quality threshold *δ*: the optimal total quality cost is declining basically with the decrease of *δ* for each selection of *T*; the optimal values with *T* = 130 and *T* = 150 are nearly consistent, and both less than that with *T* = 110 and *T* = 170. It becomes obvious that the quality cost reduces due to the lower of the quality requirement or the extension of project deadline. However, when the extended deadline surpasses a critical interval, the quality cost could not be reduced. The critical interval decides whether the extension influences on the quality cost for different project instance. The deadline *T* ∈ {130,150} is the critical interval in the paper. Thus, the timing of 130 or 150 days is the most appropriate time span for the construction project, and the threshold *δ* should be prescribed according to the cost constraint.

We further evaluate the SFLA by comparing with the objective of minimum activity quality (O = MAQ) and average activity quality (O = AAQ) for different deadline *T*. In Table 7, for the SFLA with different quality threshold *δ* ∈ {0.9,0.85,0.8}, we list minimum quality (MQ) and average quality (AQ) of all the optimal solution of Fig 6; for the O = MAQ or O = MAQ, their objective values (MQ or AQ) and total quality costs (TC) are both presented. From Table 7, all the objective value for the O = MAQ or O = MAQ are 0.95 or 0.91 respectively. Without cost constraint, they both obtain an optimal project quality. However for the SFLA with *δ* = 0.9, we could attain the similar MQ and AQ but spend much lower quality cost.

**Table 7:** Result comparison of minimum quality.

*T*	SFLA						O = MAQ		O = AAQ	
	*δ* = 0.9		*δ* = 0.85		*δ* = 0.8					
	MQ	AQ	MQ	AQ	MQ	AQ	MQ	TC($)	AQ	TC($)
110	0.91	0.95	0.89	0.93	0.87	0.92	0.91	65019	0.95	65019
130	0.9	0.94	0.9	0.93	0.86	0.91	0.91	65019	0.95	65019
150	0.9	0.95	0.9	0.93	0.86	0.91	0.91	65019	0.95	65019
170	0.91	0.95	0.89	0.93	0.86	0.92	0.91	65019	0.95	65019

## Conclusion

A new non-linear programming model based on the multi-mode resource constrained project scheduling problem was developed. The objective is to minimize the total quality cost, which consists of prevention cost and failure cost, in order to search for an optimal quality and avoid considerable trade-offs between project costs and project quality. In the model, repair works would be activated during construction if the cumulative quality of a certain activities was substandard under inspection. Moreover, activity quality and cumulative quality are illustrated specifically:

- A binary normal distribution function is adopted and evaluated to describe activity quality under the assumption that the activity duration is dependent of its direct cost. The non-independent relationship is presented by the multiple modes of each activity.
- Cumulative quality expresses the quality of a certain finished activities and may trigger repair work or rework, and it is defined by two different relationships among activity qualities: the activities bound by coordinative relationship is regarded as a prerequisite for the other activities to improve project quality, so their cumulative quality is described by an arithmetic average; activities with a precedence relationship are on an equal footing, so their cumulative quality is formulated in terms of an arithmetic product.

Furthermore, a shuffled frog-leaping algorithm is developed to solve the discrete trade-off problem. It is encoded in an adjusted activity list and decoded by an adaptive serial schedule generation scheme. The algorithm consists of four steps: population initialisation, selection, population partition and frog-leaping progression. The frog-leaping progress combines the crossover operator of genetic algorithm and a permutation-based local search to improve efficiency. Finally, a construction project for a framed railway overpass is provided to examine the algorithm performance by a convergence curve. Moreover, a sensitivity analysis and a comparison with different objectives are performed to determine the satisfactory project deadline and quality threshold and to assist the decision makers in addressing the time-cost-quality problem. In future research, we will extend this study by multiple projects to be a geographically distributed scheduling problem [23].

## Supporting Information

S1 Table Relevant data with respect to the main resources and duration. (DOCX)

S2 Table Activity qualities in different modes. (DOCX)
