# Supplementary material for: A New Model for Solving Time-Cost-Quality Trade-Off Problems in Construction
Source: PLoS One. 2016 Dec 2;11(12):e0167142. doi: 10.1371/journal.pone.0167142 (PMC5135070; doi:10.1371/journal.pone.0167142)
Supplement: S1 Table — (DOCX) [file pone.0167142.s001.docx]

**S1 Table. Relevant data with respect to the main resources and duration**

| **No.** | **Duration**  **(day)** | **Clam shell excavator** | **Hoisting jack** | **Steel bar processing machinery** | **Skilled worker** | **General**  **worker** | **bitumen 2 mm**  **(m^2^)** | **bitumen 3 mm**  **(m^2^)** |
| --- | --- | --- | --- | --- | --- | --- | --- | --- |
| 1 | 3 | 1 | 0 | 0 | 2 | 6 | 0 | 0 |
|  | 4 | 1 | 0 | 0 | 4 | 8 | 0 | 0 |
|  | 3 | 2 | 0 | 0 | 6 | 10 | 0 | 0 |
| 2 | 2 | 0 | 0 | 0 | 0 | 5 | 0 | 0 |
|  | 2 | 0 | 0 | 0 | 2 | 5 | 0 | 0 |
|  | 3 | 0 | 0 | 0 | 3 | 7 | 0 | 0 |
| 3 | 2 | 0 | 0 | 0 | 0 | 8 | 0 | 0 |
|  | 3 | 0 | 0 | 0 | 2 | 5 | 0 | 0 |
|  | 3 | 0 | 0 | 0 | 4 | 8 | 0 | 0 |
| repair | 3 | 0 | 0 | 0 | 5 | 9 | 0 | 0 |
| 4 | 25 | 0 | 0 | 3 | 2 | 24 | 0 | 0 |
|  | 26 | 0 | 0 | 3 | 5 | 20 | 0 | 0 |
|  | 25 | 0 | 0 | 5 | 9 | 24 | 0 | 0 |
| 5 | 18 | 0 | 0 | 0 | 0 | 25 | 0 | 0 |
|  | 19 | 0 | 0 | 0 | 5 | 20 | 0 | 0 |
|  | 21 | 0 | 0 | 0 | 8 | 20 | 0 | 0 |
| 6 | 28 | 0 | 0 | 0 | 10 | 40 | 0 | 0 |
|  | 30 | 0 | 0 | 0 | 20 | 40 | 0 | 0 |
|  | 29 | 0 | 0 | 0 | 30 | 40 | 0 | 0 |
| 7 | 18 | 0 | 0 | 0 | 2 | 20 | 5915 | 0 |
|  | 18 | 0 | 0 | 0 | 7 | 16 | 5915 | 0 |
|  | 22 | 0 | 0 | 0 | 7 | 16 | 0 | 5915 |
| repair | 25 | 0 | 0 | 3 | 10 | 40 | 0 | 1073 |
| 8 | 7 | 0 | 0 | 0 | 3 | 17 | 0 | 0 |
|  | 7 | 0 | 0 | 0 | 5 | 15 | 0 | 0 |
|  | 6 | 0 | 0 | 0 | 7 | 16 | 0 | 0 |
| 9 | 2 | 0 | 0 | 0 | 1 | 12 | 0 | 0 |
|  | 2 | 0 | 0 | 0 | 3 | 10 | 0 | 0 |
|  | 4 | 0 | 0 | 0 | 3 | 7 | 0 | 0 |
| 10 | 2 | 0 | 0 | 0 | 2 | 13 | 0 | 0 |
|  | 2 | 0 | 0 | 0 | 7 | 10 | 0 | 0 |
|  | 3 | 0 | 0 | 0 | 6 | 14 | 0 | 0 |
| 11 | 4 | 0 | 0 | 0 | 5 | 25 | 0 | 0 |
|  | 5 | 0 | 0 | 0 | 10 | 20 | 0 | 0 |
|  | 4 | 0 | 0 | 0 | 17 | 20 | 0 | 0 |
| 12 | 5 | 0 | 4 | 0 | 10 | 40 | 0 | 0 |
|  | 5 | 0 | 4 | 0 | 15 | 50 | 0 | 0 |
|  | 6 | 0 | 8 | 0 | 25 | 60 | 0 | 0 |
| repair | 14 | 0 | 5 | 0 | 15 | 50 | 0 | 0 |
| 13 | 4 | 0 | 0 | 0 | 0 | 30 | 0 | 0 |
|  | 5 | 0 | 0 | 0 | 0 | 50 | 0 | 0 |
|  | 4 | 0 | 0 | 0 | 10 | 50 | 0 | 0 |
| 14 | 4 | 0 | 0 | 0 | 0 | 20 | 0 | 0 |
|  | 5 | 0 | 0 | 0 | 3 | 15 | 0 | 0 |
|  | 4 | 0 | 0 | 0 | 5 | 20 | 0 | 0 |
| Resource availability | | 2 | 8 | 5 | 50 | 80 | 5915 | 7800 |
| Unit cost ($) | | 85.34 | 1.82 | 5.63 | 38.63 | 20.09 | 3.09 | 4.02 |
