# Supplementary material for: A New Model for Solving Time-Cost-Quality Trade-Off Problems in Construction
Source: PLoS One. 2016 Dec 2;11(12):e0167142. doi: 10.1371/journal.pone.0167142 (PMC5135070; doi:10.1371/journal.pone.0167142)
Supplement: S2 Table — (DOCX) [file pone.0167142.s002.docx]

**S2 Table. Activity qualities in different modes**

| **No.** | **mode** | **Activity quality** | **No.** | **mode** | **Activity quality** | **No.** | **mode** | **Activity quality** |
| --- | --- | --- | --- | --- | --- | --- | --- | --- |
| 1 | 1 | 0.88 | 6 | 1 | 0.89 | 11 | 1 | 0.87 |
|  | 2 | 0.90 |  | 2 | 0.94 |  | 2 | 0.91 |
|  | 3 | 0.91 |  | 3 | 0.98 |  | 3 | 0.93 |
| 2 | 1 | 0.86 | 7 | 1 | 0.91 | 12 | 1 | 0.88 |
|  | 2 | 0.94 |  | 2 | 0.95 |  | 2 | 0.95 |
|  | 3 | 0.97 |  | 3 | 0.97 |  | 3 | 0.97 |
| 3 | 1 | 0.89 | 8 | 1 | 0.87 | 13 | 1 | 0.86 |
|  | 2 | 0.93 |  | 2 | 0.90 |  | 2 | 0.91 |
|  | 3 | 0.96 |  | 3 | 0.92 |  | 3 | 0.94 |
| 4 | 1 | 0.93 | 9 | 1 | 0.91 | 14 | 1 | 0.90 |
|  | 2 | 0.96 |  | 2 | 0.95 |  | 2 | 0.92 |
|  | 3 | 0.98 |  | 3 | 0.95 |  | 3 | 0.93 |
| 5 | 1 | 0.86 | 10 | 1 | 0.89 |  |  |  |
|  | 2 | 0.89 |  | 2 | 0.92 |  |  |  |
|  | 3 | 0.97 |  | 3 | 0.95 |  |  |  |
